# In Situ Maturated Early-Stage Human-Induced Pluripotent Stem Cell-Derived Cardiomyocytes Improve Cardiac Function by Enhancing Segmental Contraction in Infarcted Rats

**DOI:** 10.3390/jpm11050374

**Published:** 2021-05-04

**Authors:** Diogo Biagi, Evelyn Thais Fantozzi, Julliana Carvalho Campos-Oliveira, Marcus Vinicius Naghetini, Antonio Fernando Ribeiro, Sirlene Rodrigues, Isabella Ogusuku, Rubia Vanderlinde, Michelle Lopes Araújo Christie, Debora Bastos Mello, Antonio Carlos Campos de Carvalho, Marcos Valadares, Estela Cruvinel, Rafael Dariolli

**Affiliations:** 1 PluriCell Biotech, São Paulo 05508-000, Brazil; diogo.biagi@pluricellbiotech.com.br (D.B.); efantozzi88@gmail.com (E.T.F.); julliana.carvalho@pluricellbiotech.com.br (J.C.C.-O.); mv_patela@hotmail.com (M.V.N.); antonio.ribeiro@pluricellbiotech.com.br (A.F.R.J.); sirlene.rodrigues@pluricellbiotech.com (S.R.); isabellaogusuku@gmail.com (I.O.); ruh.chuartz@gmail.com (R.V.); marcos.valadares@pluricellbiotech.com.br (M.V.); estela.cruvinel@pluricellbiotech.com.br (E.C.); 2 Gene Center and Department of Biochemistry, Ludwig-Maximilians-Universität München, 81377 München, Germany; 3 Carlos Chagas Filho Institute of Biophysics, Federal University of Rio de Janeiro, Rio de Janeiro 21941-902, Brazil; michellechristie2107@gmail.com (M.L.A.C.); debmello2003@gmail.com (D.B.M.); acarlos@biof.ufrj.br (A.C.C.d.C.); 4 Department of Pharmacological Sciences, Icahn School of Medicine at Mount Sinai, New York, NY 10029, USA

**Keywords:** stem cell-therapy, human induced pluripotent stem cells, cardiomyocytes, myocardial infarction, heart failure, regeneration, cardiac function

## Abstract

The scant ability of cardiomyocytes to proliferate makes heart regeneration one of the biggest challenges of science. Current therapies do not contemplate heart re-muscularization. In this scenario, stem cell-based approaches have been proposed to overcome this lack of regeneration. We hypothesize that early-stage hiPSC-derived cardiomyocytes (hiPSC-CMs) could enhance the cardiac function of rats after myocardial infarction (MI). Animals were subjected to the permanent occlusion of the left ventricle (LV) anterior descending coronary artery (LAD). Seven days after MI, early-stage hiPSC-CMs were injected intramyocardially. Rats were subjected to echocardiography pre-and post-treatment. Thirty days after the injections were administered, treated rats displayed 6.2% human cardiac grafts, which were characterized molecularly. Left ventricle ejection fraction (LVEF) was improved by 7.8% in cell-injected rats, while placebo controls showed an 18.2% deterioration. Additionally, cell-treated rats displayed a 92% and 56% increase in radial and circumferential strains, respectively. Human cardiac grafts maturate in situ, preserving proliferation with 10% Ki67 and 3% PHH3 positive nuclei. Grafts were perfused by host vasculature with no evidence for immune rejection nor ectopic tissue formations. Our findings support the use of early-stage hiPSC-CMs as an alternative therapy to treat MI. The next steps of preclinical development include efficacy studies in large animals on the path to clinical-grade regenerative therapy targeting human patients.

## 1. Introduction

Cardiovascular diseases, in particular myocardial infarction (MI), are the leading cause of morbi-mortality worldwide [1,2]. The adult heart displays a limited regenerative capacity due to the scant ability of cardiomyocytes to proliferate [3,4]. Therapies currently available partially preserve the heart’s structure and function without any sign of muscle regeneration. In this scenario, patients with decompensated outcomes ultimately experience severe heart failure conditions requiring heart transplantation [5].

Alternatively, stem cell-based therapies have been proposed to overcome the lack of heart re-muscularization within the available therapeutics [5]. Remarkably, multipotent stem cells isolated from adult tissues such as bone marrow [6,7,8,9,10] and adipose tissue [11,12] have demonstrated unsatisfactory heart regeneration performance. Conversely, human pluripotent stem cells such as embryonic stem cells (hESCs) [13] and induced pluripotent stem cells (hiPSCs) [14,15] are promising sources for the generation of cardiomyocytes (hPSC-CMs) in vitro [16,17]. Contractile hPSC-CMs are immature compared with adult cardiomyocytes [18,19,20], a double-edged characteristic for cell therapy. These cells display proliferation capacity, which is gradually lost as the maturation level increases over time in vitro and in vivo [18,21,22]. Nevertheless, hPSC-CMs reaching adult cardiomyocyte levels have not been reported upon yet.

Independently of this limitation, several groups have demonstrated that the transplantation of hPSC-CMs enhances cardiac function through human cardiac tissue engraftment [21,23,24,25,26,27,28,29,30,31,32,33,34]. Despite promising results, the perfect interaction between host and graft (e.g., the mitigation of arrhythmias), satisfactory graft maturation levels over time in vivo, and other fundamental questions such as cell dose, hiPSC-CM age at the time of injection, delivery method, and the degree of importance of each of these factors for a significant and sustained structural and functional improvement, remain elusive.

In the last decades our group has been working to develop stem cell-based therapies to treat myocardial infarction building tools [35,36,37,38] and testing trending therapies [9,11,12,39,40] always aimed at patients [6,7,8]. Adult stem cell approaches to treat MI have yet to yield relevant results clinically [41] and it is generally accepted that other approaches are needed [42]. Here, we demonstrate that early-stage hiPSC-CM injections enhance the overall cardiac function of infarcted rats under immunosuppression. Treated rats had their cardiac function improved due to the efficient engraftment of the human cardiac cells onto the fibrotic tissue. Moreover, human cardiac grafts showed enhanced maturation in situ, preserving some degree of proliferation capacity. Furthermore, our grafts were vascularized by host vessels and did not elicit significant immune rejection. These findings support further studies in larger animal models to conquer the limitations inherently present in a rodent cardiovascular system, with the aim to develop scalable and efficient regenerative stem cell-based therapies for humans.

## 2. Results

### 2.1. Descriptive Analysis of Mortality and Echocardiography-Based Randomization

Forty-two female rats were used in this study. Thirty-eight animals were subjected to a surgical thoracotomy for MI induction (33 MIs and 5 SHAM-operated rats), four rats were used as healthy controls. Notably, only one rat died during MI induction and two others within the first six days after the procedure (Table 1). From forty-eight hours after MI until the end of the protocol (37 days after MI), the SHAM and MI-induced rats received two daily doses of cyclosporine A (CsA). CsA toxicity was assessed by monitoring the body mass of the animals weekly. Body mass changes were similar between groups over time (Figure S1A). Six days after MI induction, 39 rats (4 CTRLs, 5 SHAMs, and 30 MIs) were subjected to baseline echocardiography to assess left ventricle (LV) cardiac function. Two infarcted animals died due to anesthetic overload during the echocardiography (Table 1). The LV ejection fraction (LVEF) of each animal was estimated. LVEF values were used to randomize infarcted animals into two balanced groups: (1) PSC (placebo, received injections of pro-survival cocktail supplemented vehicle, 14 animals) and (2) CELL (PSC vehicle and approx. 10 million hiPSC-CMs, 14 animals) (Table S1).

**Figure 1:**
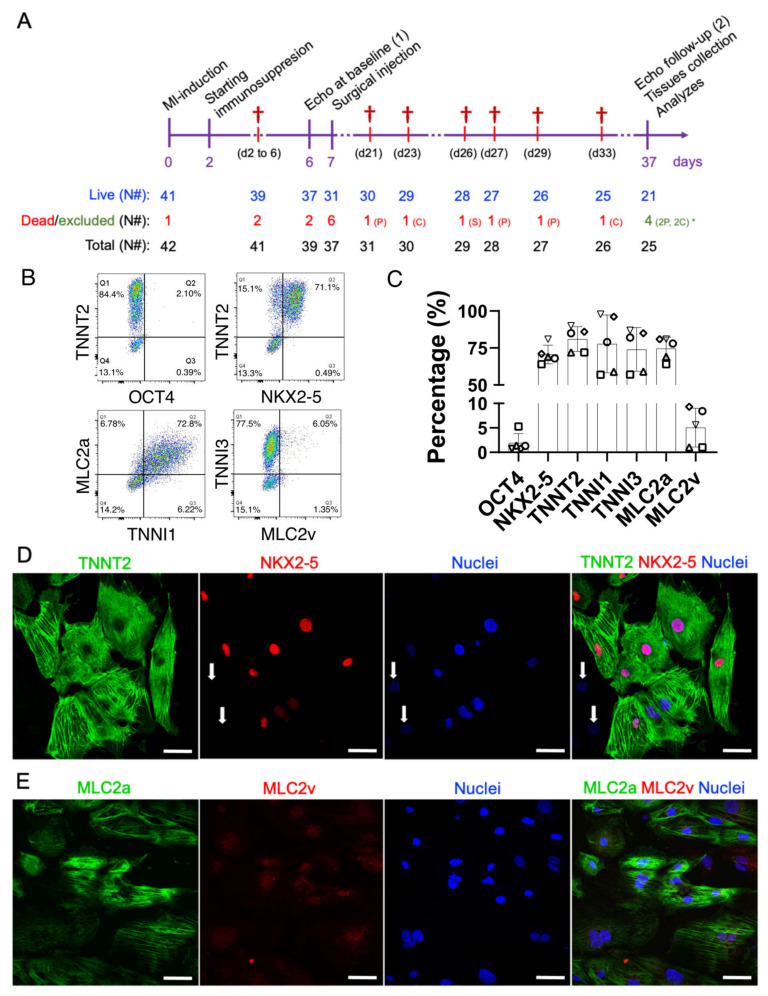
Therapeutic study design and molecular characterization of the early-stage hiPSC-CMs. (**A**) On day zero, myocardial infarctions were induced by the permanent occlusion of the proximal AD coronary artery. Forty-eight hours after MI (day 2), the immunosuppression protocol was started. CsA was administered twice a day intraperitoneally until the end of the protocol (day 37). Six days after the MI induction (day 6), all animals were subjected to baseline echocardiography and randomized. On day 7, animals from placebo (pro-survival cocktail—PSC) and CELL groups were subjected to a second surgical thoracotomy followed by the intramyocardial injection of hiPSC-CMs. Thirty days after injection (37 days after the MI induction), all survivors were subjected to a final echocardiography and were then euthanized, necropsied, and had their hearts and other organs collected and stored for further analysis. (**B**) Representative scatterplots of the injected hiPSC-CM population on days 11–15 of differentiation. (**C**) The number of positive cells expressing cardiac markers such as TNNT2, TNNI1, TNNI3, MLC2A, MLC2V, and NKX2-5, and a pluripotency marker OCT4, was obtained by flow cytometry, expressed as a percentage, and plotted in bar charts (mean ± SD). (**D**) Representative immunofluorescence demonstrating the expression of cardiac markers in hiPSC-CMs on day 13 of differentiation (TNNT2, NKX2-5, and nuclei). Except for two cells in the image (white arrows), all the TNNT2-positive cells were also NKX2-5 positive. (**E**) MLC2a, MLC2v, nuclei, and merged image. Note that the expression of MLC2v seems to be nuclear/perinuclear, strongly corroborating the lack of maturation of early-stage hiPSC-CMs. Scale bars: 100 µm.

Seven days after MI induction, PSC and CELL rats were subjected to a second surgical thoracotomy for cell transplantation (or placebo). Six rats (three PSC and three CELL) died during the intramyocardial injection surgery (Table 1). The thirty-one remaining rats were followed for 30 days after injections (Figure 1A). Throughout the four weeks one SHAM animal died on day 19; three PSC rats died on days 14, 20, and 22; and two CELL animals died on days 16 and 26 after injection procedure (Table 1). Mortality rates were not significantly different between groups after treatment (Figure S1B). Finally, two PSC and two CELL rats were excluded from further comparative analyzes since they displayed less than 20% impairment of LVEF compared to healthy animals at baseline. Thus, all analyzes were performed comparing four CTRL, four SHAM, six PSC, and seven CELL animals. The therapeutic protocol adopted is graphically represented in Figure 1A.

### 2.2. Early-Stage Human iPSC-CMs Are Predominantly MLC2a Positive (Atrial-Like Cardiomyocytes) at the Time of Injection

Seven days after the MI induction PSC injections (placebo), or 10 million early-stage hiPSC-CMs in the range of 11–15 days of differentiation, were subjected to heat-shock priming [23,27] and intramyocardially injected (from epi- to myocardium) into three different points of the cardiac scar of the MI-induced rats. Aliquots of the injections were collected and characterized by flow cytometry and immunofluorescence (Figure 1B–E). All animals received at least 65% of NK2 Homeobox 5 (NKX2-5)/Troponin T2 (TNNT2) positive cells (70.7 ± 5.7% and 81 ± 8.4%, Figure 1B–D). Furthermore, these cells expressed Troponin I1 (TNNI1, 77.8% ± 19.6) and Myosin light chain 2, atrial isoform (MLC2a, 74.6 ± 7.7%), both markers characteristic of early-stage hiPSC-CM (Figure 1B,C). Despite their intrinsic immaturity, these cardiomyocytes also expressed Troponin I3 (TNNI3, 74 ± 14.7%) (Figure 1B,C). As expected, MLC2v (ventricular myosin light chain 2) was detected in a relatively small percentage of the population (5.1 ± 4%) (Figure 1B,C,E), also evidenced by immunofluorescence imaging (Figure 1D,E). Finally, less than 2% of the injected cells expressed OCT4 (1.9 ± 1.9%) (Figure 1B,C). Altogether, these findings demonstrate that all the animals were treated with a homogeneous population of hiPSC-CMs with very low ranges of non-myocyte cells.

### 2.3. Early-Stage Human iPSC-CM Therapy Significantly Improves the Overall Cardiac Function of Immunosuppressed Infarcted Rats

**Figure 2:**
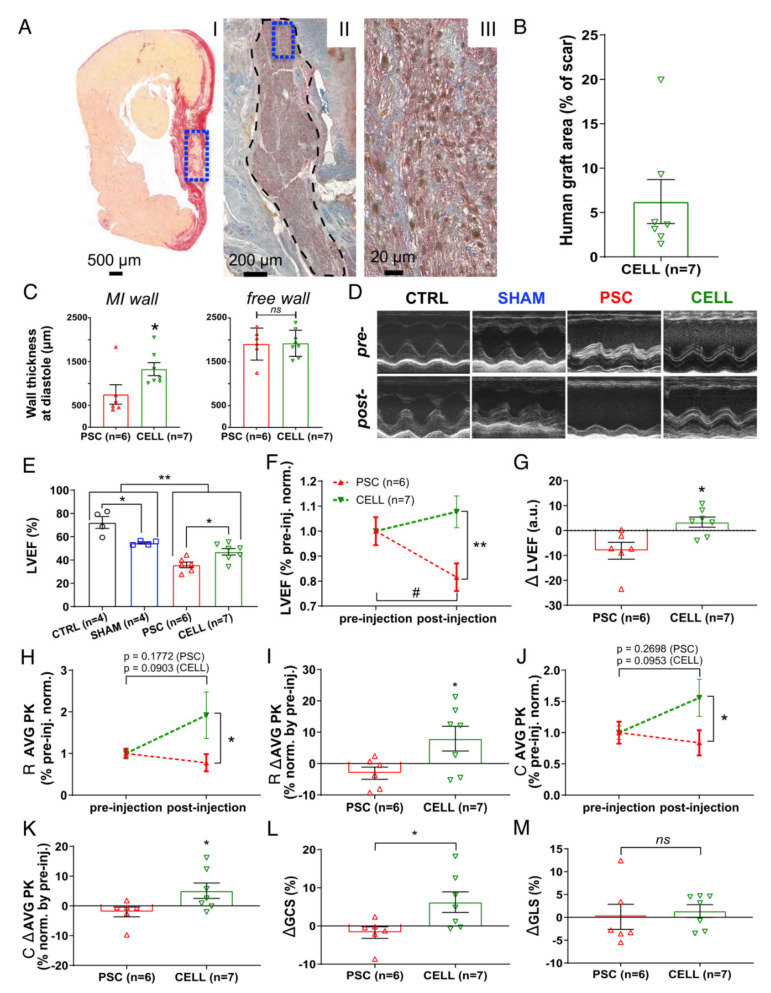
Early-stage hiPSC-CM-based therapy improves segmental contraction resulting in overall LV cardiac function preservation on infarcted rats. Echocardiography was performed on days 6 (1 day prior to injections) and 37 (30 days after injection) to evaluate the cell therapy effects. (**A**) Representative panel of histological images from animal 29 (CELL group). (AI) Papillary level cross-section stained with picrosirius red evidencing “live” cardiac tissue (yellow) and fibrosis (red). “Live” cardiac tissue was found in the scar (blue box). (AII) Subsequent cross-section subjected to an immunohistochemical reaction against human Ku80 (brown nuclei) and Troponin T2 (red), evidencing human cells in the rat heart (black dashed area). (AIII) The human cardiac graft is taken from positive cells for both markers (brown nuclei—hKu80, and red fibers—Troponin T2) (From blue box in AII). (**B**) Percentage of the area covered by hKu80-positive cells in the scar tissue was quantified in 3–5 cross-sections at the papillary level. The human graft areas were expressed as a percentage relative to the scar area. All the CELL-treated rats displayed hKu80/Troponin T2-positive areas. (**C**) Wall thickness at diastole was measured at the papillary level using low magnification images. MI (left chart) and free wall (right chart) values were calculated as a mean of three measurements covering the whole scar extension and expressed as μm. (**D**) Representative short-axis of M-mode images pre- (day 6) and post-injection (day 37). (**E**) LVEF was calculated using short-axis images at four LV depths by a modified Simpson algorithm. CELL-treated rats showed improved LVEF 30 days post-treatment (vs. PSC, * *p* < 0.05). Furthermore, MI-induced animals showed significantly lower LVEF values than the CTRL and SHAM groups (** *p* < 0.01). Additionally, the CTRL and SHAM groups displayed different LVEF values (* *p* < 0.05). (**F**) The LVEF values of PSC and CELL rats were normalized by their respective pre-treatment values and plotted over time. CELL-treated rats show higher LVEF values than PSCs post-treatment (** *p* < 0.01). PSCs showed a significant deterioration of LVEF 37 days after MI-induction (# *p* = 0.0145), whereas CELL rats had their LVEF preserved (*p* = 0.2162). (**G**) The difference in LVEF values between pre- and post-injection (delta) shows cardiac function preservation in the CELL group (* *p* = 0.0117 vs. PSC). (**H**) Radial strain time-to-peak (AVG PK) of PSC and CELL rats were normalized by their respective pre-treatment values. The CELL group displayed higher radial strain values AVG PK post-treatment (* *p* = 0.0441). Pre- vs. post-treatment analysis revealed no significant differences in the PSC nor CELL group, as demonstrated by the *p*-values printed in the image. (**I**) Radial strain time-to-peak values between pre- and post-injection (delta) were also significantly different (* *p* < 0.0371). (**J**) Circumferential strain time-to-peak (AVG PK) values of the PSC and CELL rats were normalized by their respective pre-treatment values. The CELL group displayed higher circumferential strain AVG PK post-treatment (* *p* = 0.0494). Pre- vs. post-treatment analysis revealed no significant differences in the PSC nor CELL group, as demonstrated by the *p*-values printed in the image. (**K**) The circumferential strain time-to-peak delta was also significantly different (* *p* = 0.0465). (**L**) The global circumferential strain between pre- and post-injection (delta) was significantly positive for CELL-treated animals (*p* = 0.0324 vs. PSC), but (**M**) the global longitudinal strain between pre- and post-injection (delta) did not display changes (data positively normalized; *p* = 0.6767 CELL vs. PSC). Data showed as mean ± SE. R: radial; C: circumferential; GCS: global circumferential strain; GLS: global longitudinal strain.

Four weeks after injections, grafts of human cardiac tissue were found in the LV of the animals treated with the hiPSC-CM therapy (Figure 2A). The mean percentage of the human graft was 6.23% ± 2.29% (human Ku80 (hKu80)/TNNT2-positive cells (rat and human marker) in the fibrotic tissue, Figure 2B and Table S2). Interestingly, hiPSC-CM-treated rats displayed significantly thicker MI-affected walls (* *p* < 0.05 vs. PSC, Figure 2C, left bars). Conversely, we did not find any difference in the thickness at free walls (Figure 2C, right bars). However, the percentage of the injured area did not change on the CELL group (vs. PSC rats, *p* = 0.47, Figure S2A).

Moreover, the significant impairment of cardiac function at baseline in infarcted rats (PSC and CELL) was shown, as evidenced by lower values of LVEF (vs. CTRL and SHAM animals, Figure S2B). In addition, the PSC and CELL groups exhibited similar LVEF values pre-treatment (Figure S2B). Nevertheless, 30 days after treatment, rats from the CELL group showed higher LVEF values (vs. PSC group; Figure 2D,E). Indeed, most of the rats treated with the placebo demonstrated a significant deterioration in their cardiac function (Figure 2F; # *p* < 0.05 vs. pre-treatment, and Figure S2C). Conversely, hiPSC-CM-treated animals had their LVEF values preserved over time (Figure 2F; ** *p* < 0.05 vs. PSC, and Figure S2D). The benefit of the cell therapy was evidenced through the positive LVEF delta calculated by the pre- and post-treatment LVEF values (* *p* < 0.05, Figure 2G).

Besides the improvement in LVEF values, the radial (* *p* < 0.05, Figure 2H) and circumferential (* *p* < 0.05, Figure 2J) strain time-to-peak (AVG PK), calculated by the average of six segments measured in short-axis images at the papillary level, were significantly higher in the hiPSC-CM-treated rats than in the PSC group (individual curves for each animal are shown in Figure S3). In addition, both radial and circumferential strain time-to-peak deltas (* *p* < 0.05, Figure 2I,K respectively) and global circumferential strain (Figure 2L) were significantly positive on the CELL-treated animals. Conversely, none of the previous parameters changed significantly for the radial and longitudinal strain as calculated using long-axis images (Figure 2M and Figure S4). Overall, the speckle tracking analysis corroborates the LVEF improvement, strongly suggesting enhancements in segmentary contraction supporting the active human graft presence into the mid-portion of the rat’s LV walls.

Despite the improvement in cardiac function observed in the CELL group, the LVEF values of the treated rats were still significantly smaller than those observed in non-infarcted animals (CTRLs and SHAM) (Figure 2E).

### 2.4. The Human Grafts Are Composed of Cardiomyocytes That Preserve Certain Levels of Cell Cycling Activity

**Figure 3:**
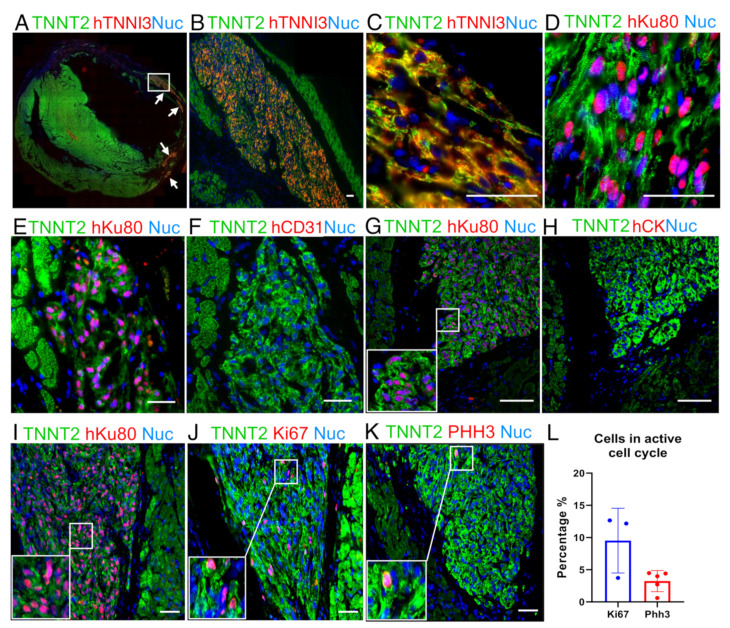
Representative image from a human graft showing cardiac cellular composition. (**A**) Panoramic immunofluorescence staining for TNNT2 (green, rat and human) and human TNNI3 (red, human-specific): arrows indicate grafts. (**B**) An amplified view of largest graft region marked by the white rectangle in A. (**C**) Higher magnification of (**B**) showing sarcomere structure organization. (**D**) Image showing that hiPSC-CMs follow cardiac structure organization, TNNT2 (green) and hKu80 (pink) by overlap for hKu80 (red) and nuclei (blue). (**E**–**H**) Images showing no evidence of hiPSC-CMs trans-differentiation into cell types other than cardiomyocytes. Serial slices were labeled for hKu80 (**E**) and human CD31 (**F**). The same strategy was used to label hKu80 (**G**) and human Cytokeratin-1 (**H**). (**I**–**L**) Ki67 and PHH3 markers evidenced that the human cardiac grafts were composed of hiPSC-CMs with an active cell cycle. (**I**) Serial slides were labeled as TNNT2 (green) and hKu80 (pink), (**J**) TNNT2 (green) and Ki67 (pink)**,** and (**K**) TNNT2 (green) and PHH3 (pink). Pink represents the overlap of red and blue (nuclei). (**L**) Quantification for cell-cycle markers presents a percentage of Ki67-positive cells and PHH3-positive cells (vs. the total number of hKu80-positive nuclei for each image analyzed). Ki67: *n* = 3 and PHH3: *n* = 5. Scale bars: 50 µm.

**Figure 4:**
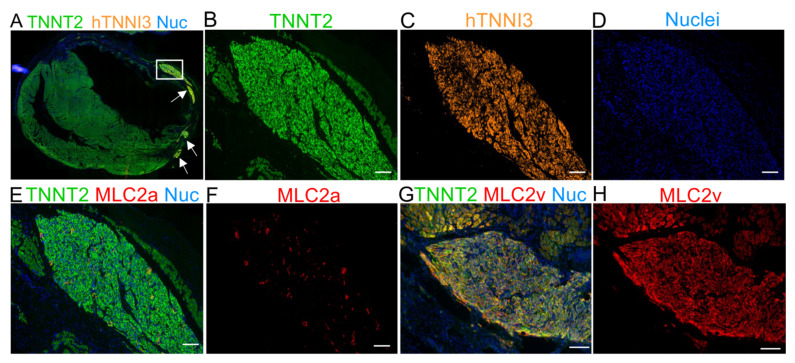
Immunofluorescence staining for MLC2a and MLC2v 30 days after cell transplantation demonstrating the maturation of hTNNI3-positive cells in vivo. (**A**) Panoramic immunofluorescence staining for TNNT2 (human and rat cardiomyocytes) and hTNNI3 (human-specific cardiomyocytes). (**B**–**D**) Images showing the area depicted by the white rectangle in A (hTNNI3-positive cardiomyocytes) in higher magnification with a single channel for each marker. (**B**) TNNT2 (green, rat and human cells), (**C**) human TNNI3 (orange, note the absence of rat tissue compared with (**B**,**D**) nuclei (blue). (**E**) Serial slices were stained with TNNT2 (green), MLC2a (red), and nuclei (blue). Note that the graft in C is the same as that used in E. (**F**) MLC2a (red) showing scant expression on the graft. (**G**) Merged image for TNNT2 (green), MLC2v (red), and nuclei (blue). (**H**) MLC2v showing extensive labeling, compared with MLC2a in (**F**). Scale bars: 100 µm.

**Figure 5:**
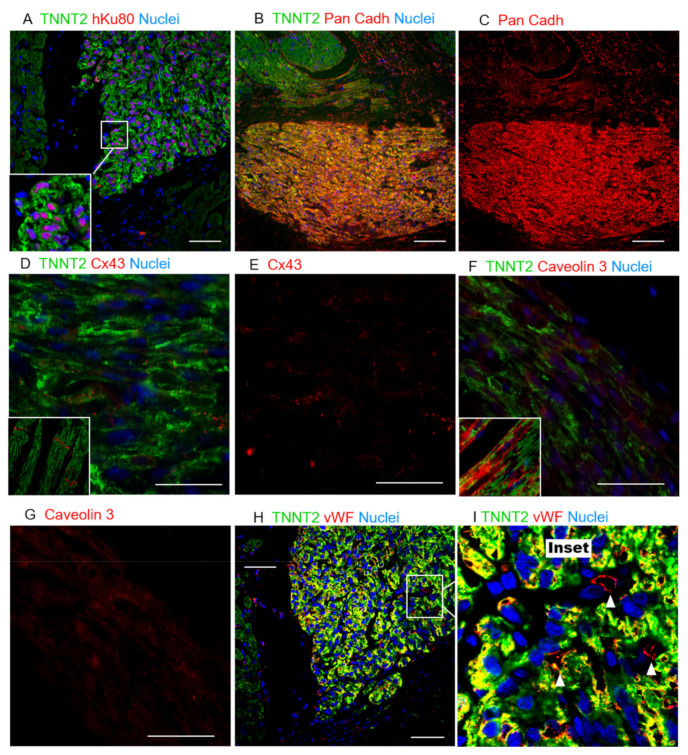
Histological evaluation of hiPSC-CM grafts suggesting limited host/graft interaction. (**A**) Colocalization of hKu80 (red) and nuclei (blue), and TNNT2 (green). (**B**) Pan-cadherin expression in both the host and human grafts indicates a high cell-to-cell interaction. (**C**) Pan-cadherin is more highly expressed on the human cardiac graft than on the host tissue. (**D**) The expression of Cx43 is evidently lower and more randomly distributed across the human cardiac cells, whereas a structured expression can be observed in the host tissue (inset), particularly on the short-borders of adult cardiomyocytes. (**E**) Cx43 single-channel image. (**F**) Caveolin3 (red) was also found in grafts at lower levels of expression and organization than in adult cardiomyocytes, where this protein is structurally expressed inside the *caveolae* within the T-tubules (inset), suggesting hiPSC-CMs limited maturation. (**G**) Cav3 single-channel image. (**H**,**I**) Rat vWF-positive blood vessels (red) perfusing the human cardiac grafts, observed in abundance in all the animals tested. (**I**) A magnification of H: white arrowheads show individual vWF-positive vessels (red stain). Scale bars: 50 µm.

Since the animals treated with early-stage hiPSC-CMs showed a significant improvement in their cardiac function, we assessed the human cardiac grafts’ cellular composition (Figure 3), cell cycle characteristics (Figure 3), and maturation levels (Figure 4 and Figure 5).

Overall, all the grafts identified were found in the myocardium, with most of them appearing in the scar area (Figure 3A). These human tissues displayed a highly compacted structure and a very distinct morphology compared to the host tissue (Figure 3B). Of note, even using standard staining methods alone (e.g., hematoxylin and eosin—H&E), large grafts could be identified due to their distinct morphology. Besides the hKu80/TNNT2 expression (Figure 2A and Table S2), the human grafts sizably expressed human TNNI3 (Figure 3A–C, Figure S5A–C). Additionally, structured sarcomeres were found in most of the grafts (Figure 3C,D and Figure S5B). Furthermore, we did not find evidence for human cell trans-differentiation into other cell types such as endothelial (human platelet endothelial cell adhesion molecule (PECAM-1 or CD31) positive, Figure 3E,F and Figure S5E) or epithelial cells (human Cytokeratin-1—CK, positive, Figure 3G,H and Figure S5D).

Adult cardiomyocytes display scant proliferation capacity [43,44]. On the other hand, previous reports have demonstrated that hiPSC-CMs can proliferate in vitro and in vivo [21,34]. Ki67 and Phosphohistone H3 (PHH3) are widespread markers of cells in the active cell cycle [45]. We used sequential slides of hKu80-positive grafts (Figure 3I) to assess Ki67 (Figure 3J) and PHH3 (Figure 3K) expression in the human grafts. The percentage of Ki67-positive nuclei in the hKu80 grafts was 9.52 ± 5.02% (mean ± SD) (Figure 3J,L). In addition, the percentage of PHH3-positive nuclei in the hKu80 grafts was 3.24 ± 1.64% (mean ± SD) (Figure 3K,L).

### 2.5. The Human Cardiac Grafts Maturate In Situ after Injection

hiPSC-CMs are immature compared to adult cardiomyocytes [19,20]. Previous reports have used protein expression shifts (e.g., TNNI1 to TNNI3, MLC2a to MLC2v) to monitor the increasing levels of maturation of hiPSC-CMs in vitro and in vivo [22,34]. We used sequential slides of human TNNI3 (hTNNI3)/TNNT2 (rat and human) positive tissues (Figure 4A–D) to evaluate MLC2a (Figure 4E,F) and MLC2v (Figure 4G,H) expression in the human grafts. Approximately 30 days after the cell transplantation, the expression of MLC2a, which is highly expressed in the cells on days 11–15 of differentiation in vitro (Figure 1E), was almost undetectable in vivo (Figure 4E,F and Figure S6A). Conversely, the expression of MLC2v was highly enhanced in vivo (Figure 4G,H vs. Figure 1E, and Figure S6B).

Additionally, the expression of Pan-cadherin was identified in the human cardiac grafts and host tissue, supporting cell–cell interactions in the grafted tissue (Figure 5A–C and Figures S6C and S7). Despite the evidence of maturation and interactions between the hiPSC-CMs in vivo, the expression of Connexin 43 (Figure 5D,E and Figures S6D and S8) and Caveolin 3 (Figure 5F,G and Figures S6E and S9) proteins, two other crucial markers that support higher levels of cardiac-tissue maturation, were scant and randomly distributed across the hKu80-positive grafts and host/graft borders. Conversely, adult myocytes expressed these proteins in a structured manner, with well-delimited expression in the connective short-borders of each cell–cell contact, and within the T-tubules, respectively (Figure 5D–G and Figure S6D,E). In parallel, we tracked the maturation of the hiPSC-CMs in 30- and 50-day cells in vitro. Interestingly, the expression of MLC2a was never down-regulated in the culture. Furthermore, MLC2v and Caveolin 3 expressions were up-regulated, but the expression of Cx43 was not significantly boosted after 50 days in the culture (Figure S10). Besides the expression of these markers, host-specific von Willebrand factor (vWF)-positive blood vessels were found in the human cardiac grafts and surrounding boundaries, strongly suggesting the host perfusion of these grafts (Figure 5H,I and Figure S6F).

Altogether, the shift between MLC2a and MLC2v strongly suggests that (1) injected immature hiPSC-CMs matured over time in vivo; (2) with the expression of Pan-cadherin suggesting an interaction between the hiPSC-CMs composing the grafts; however (3) the scant expression of Connexin-43 (Cx43) and Caveolin-3 (Cav3) is strong evidence that the cells in the grafts, during the time frame of this analysis, did not reach the desired maturation levels to mimic the adult cardiac tissue. Furthermore, despite the limited maturation on the grafted tissues, (4) these xenografts were incorporated into the infarcted heart receiving perfusion from host blood vessels.

### 2.6. Immunosuppression Confidently Preserves Human Cardiac Grafts from Rejection or Ectopic Cellular Formations

In addition to the presence of human cardiac tissue on infarcted hearts and the functional improvements observed in these rats, we evaluated whether inflammatory cells could surround human grafts as an additional metric to estimate human graft losses due to immunological rejection, despite active immunosuppressive protocol.

**Figure 6:**
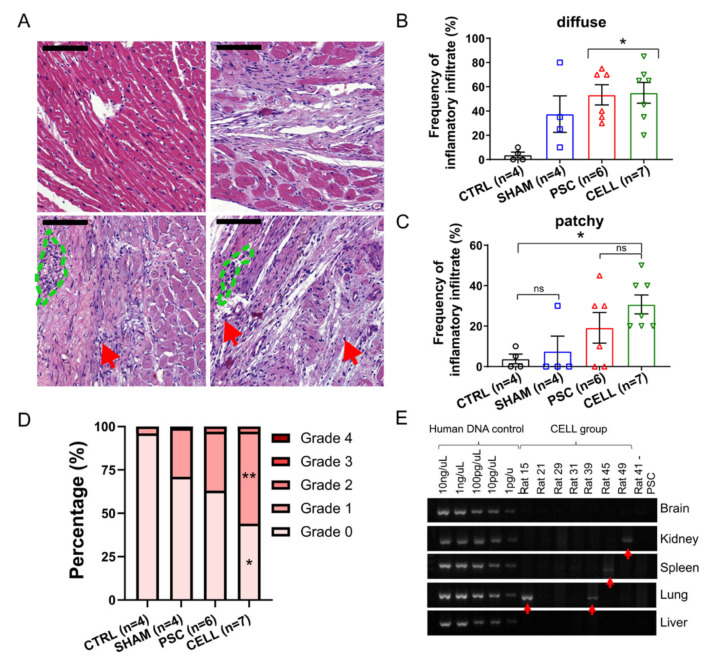
Immune-rejection against human cardiac grafts and the biodistribution of hiPSC-CMs over the body. Pathological assessments were performed through H&E-stained slides to quantify the incidence, distribution, and severity of inflammatory infiltrates. Representative images of heart sections for (**A**) CTRL, SHAM, PSC, and CELL rats (left to right, top to bottom). Red arrows indicate sparse diffuse inflammatory cells, and the blue dashed lines indicate small patches of inflammatory cells (scale bar: 100 µm). The frequency of (**B**) diffuse and (**C**) patchy infiltrates was quantitatively measured and expressed as a percentage. (**D**) The severity of the inflammatory infiltrate was estimated using a well-established grade system by observing the size and spread of inflammatory cells in the sections assessed. Grades were transformed into a percentage of severity per image and therefore per animal and were plotted as stacked percentage bars. Grade 2 areas were observed only in infarcted animals. Grades 0 and 1 estimations were significantly different between the CTRL and CELL groups (* *p* < 0.05, and ** *p* < 0.01, respectively). (**E**) PCR results for human mitochondrial DNA indicate the presence of human material in rat 15 (lung), rat 39 (lung), rat 45 (spleen), and rat 49 (kidney), shown by the red arrows.

A histopathological analysis using H&E-stained slides was performed to assess cellular inflammatory infiltrate incidence, distribution, and severity [12,46]. As expected, the frequency of observation for inflammatory cells in the myocardium was high in both infarcted groups (PSC and CELL, Figure 6A–D). Notably, inflammatory infiltrates were also observed in SHAM animals, but in lower frequencies than in infarcted rats (Figure 6A–D). Overall, the most frequent infiltrates were diffuse, where sparse cells were observed solely or in very small groups (5–10 cells, Figure 6A red arrows), usually near to blood vessels (Figure 6A,B). Patches of the cell infiltrate, where inflammatory cells can be seen in larger groups, were also observed but with less frequency (Figure 6A, green dashed areas, and C). Despite the incidence of inflammatory infiltrates, the degree of severity of these infiltrates was not significantly high (most of them were classified as grades 0 and 1, Figure 6D). Statistical differences were observed between the CTRL and CELL groups for the percentage of incidences of Grades 0 and 1 infiltrates, but no differences were observed when comparing CELL-treated animals with SHAM or PSC rats (Figure 6D). Besides the histopathological assessment, we performed immunohistochemistry reactions using antibodies specific to CD3, CD45, and CD20 in 3–5 papillary level cross-sections. As expected, only scant diffuse cells or very small patches could be found in the hearts of some of the SHAM (CD3: 2/4, CD45: 1/4, CD20: 2/3), PSC (CD3: 1/6, CD45: 2/6, CD20: 3/6), and CELL (CD3: 0/7, CD45: 5/7, CD20: 4/7) animals (Figure S11).

Finally, the biodistribution of the hiPSC-CMs to the kidney, lung, spleen, and brain, was evaluated to estimate harmful outcomes related to the presence of these cells out of their target organ and potential tumorigenic outcomes. Human mitochondrial DNA presence was assessed with a two-step approach [26]. In the first screening using an end-point PCR, most of the samples were negative to human DNA contamination, except for in the lungs of animals 15 and 39, the spleen of animal 45, and the kidneys of animal 49 (Figure 6E). The contaminated samples and two additional samples from different pieces of each tissue were subjected to a quantitative PCR. This analysis revealed minimal amounts of human DNA in the two lung samples from animal 15 (0.02267% and 0.00195% human DNA to total DNA, the standard curve in the Figure S12). Additionally, no macroscopic abnormal cell mass formations were found in these tissues (Figure S13).

These findings (1) corroborate the efficiency of the immunosuppression using cyclosporine A (CsA) and (2) suggest that the injection of hiPSC-CMs into the heart is safe since no abnormal formations nor (3) substantial human DNA were found in organs other than the heart.

## 3. Discussion

A rising number of pluripotent stem cell-based therapies to treat myocardial infarction have been proposed. However, only marginal improvements in cardiac function have been reported from rodents to large animal models [47,48,49]. Here, we demonstrated (1) that the intramyocardial injection of 10 million early-stage hiPSC-CMs seven days after MI improved LV segmental performance on infarcted rats. In addition, our findings show that (2) the human grafts massively expressed cardiomyocyte-related proteins, (3) with special emphases to the accentuated shift between MCL2a and MLC2v suggesting robust maturation in situ and (4) host/graft interactions and perfusion, (5) in the absence of substantial immune rejection.

Adult cardiomyocytes display a minimal regenerative capacity [43,44]. Conversely, hiPSC-CMs are immature compared to adult myocytes [19,20], preserving some capacity to proliferate in vitro and in vivo [21]. This ability is remarkably reduced over time as increasing levels of cellular maturation occur [21,34,50,51]. Furthermore, previous reports already demonstrated that day 20 hiPSC-CMs resulted in larger human cardiac grafts in the heart of infarcted mice than the injection of hiPSCs (day 0), mesoderm progenitors (day 4), cardiac progenitors (day 8), or later-stage hiPSC-CMs (day 30). Based on these previous findings and inspired by the successful work developed by Charles Murry’s group in the last decades [23,26,27,28,29,30,32], we injected heat-shock-primed [23] early-stage hiPSC-CMs (on days 11–15 of differentiation), diluted into a pro-survival cocktail [27] and supplemented with GelTrex intramyocardially, seven days after MI-induction. The priming of hPSC-CMs with heat-shock and the injection of them in a pro-survival solution has been considered standard since Murry and colleagues demonstrated these procedures combined, significantly increasing human cardiac graft sizes and hPSC-CMs survival in the heart [23,27]. In addition, the injection of a pro-survival solution in the absence of cells does not improve cardiac function [28].

In contrast to previous reports which injected cells in very acute (minutes-to-4 days after MI) [21,22,27,31,32,51] or chronic phases (4 weeks after MI) [28] of the MI, here we tested hiPSC-CM therapy in a less inflammatory but still proliferative healing phase (7 days after MI). Endogenous repair mechanisms such as angiogenesis are active at this stage, favoring cell engraftment and viability [52,53,54]. On days 11–15 of differentiation, early-stage hiPSC-CMs mainly expressed progenitors (e.g., NKX2-5) and early-stage cardiomyocyte markers (e.g., TNNI1, MLC2a), but did not express MLC2v, a proxy of more mature ventricular-like cells. Thirty days later, we found human cardiac grafts in all of the injected rats averaging across 6.23% ± 2.29% of the scar, with a maximum size of approximately 20%, twice more than demonstrated by Laflamme et al. 2007 [27] and similar to the findings of Fernandes et al. in 2010 in a chronic model of ischemia/reperfusion [28].

Our rats were subjected to echocardiography pre- and 30 days post-injections. We demonstrated that human cardiac grafts positively affect the LV function of CELL-treated rats by increasing LVEF values by 7.8% at the end of the protocol. In contrast, the PSC group displayed an 18.2% deterioration over time. Previous authors have reported functional improvements using hESC-CMs and hiPSC-CMs acutely (minute-to-4 days after MI) in rodent models. Laflamme et al. demonstrated that the LV fractional shortening (LVSF) of rats treated with hESC-CMs was preserved four weeks after treatment [27]. Fernandes et al. showed an enhancement of approximately 28% on LVSF after a 4-week treatment of infarcted rats with hESC-CMs [32]. Furthermore, in mice models of MI, Citro et al. showed a 100% increase of LVSF and LVEF after four weeks of treatment compared to placebo [31], and Saludas et al. showed an increase of approximately 25% on LVEF 2 months after cell injection [51]. On the other hand, hESC-CM therapy to treat chronic MIs (injections 1 month after MI induction) seems ineffective in improving LV function in rodents [28].

Despite these exciting results, MI often affects specific heart segments resulting in the hyperactivity of adjacent walls to compensate for muscle deterioration [43]. Measuring LV function simply by linear features may result in misinterpretation. All the reports discussed above provided echocardiographic results based on linear measurements. To avoid misinterpretation, we calculated LVEF using a modified Simpson algorithm, which considers four cross-sections of the LV to estimate volumetric changes pre-and post-treatment. Due to the higher quality of our analysis, we can conclude that our treatment resulted in a more realistic picture of the improvement in cardiac performance than previous reports.

In our study, human grafts were predominantly found in the lateral and anterior walls of the LV, mostly at the papillary level. This observation prompted us to investigate whether the overall cardiac performance enhancement is associated with segmental improvement due to human grafts. We assessed segmental contraction by speckle tracking echocardiography. Radial and circumferential strain calculated at the papillary level in the B-dimensional dynamic short-axis images were increased by 92% and 56%, respectively, in the CELL group. Conversely, PSC rats displayed a 22% and 17% deterioration of these parameters. Nevertheless, the radial and longitudinal strain, which are calculated through long-axis images incorporating segments where human grafts were not found, did not change after treatment. Altogether, these data corroborate that segmental improvement occurred due to the presence of human cardiac grafts in the mid-portion of the myocardium on CELL-treated rats.

Additionally, we investigated the composition, proliferation capacity, maturation, and graft/host tissue interactions using immunofluorescence analysis. One month after injection, the hiPSC-CM grafts identified by hKu80 or hTNNI3 expression solely displayed the expression of cardiac markers (e.g., TNNT2, TNNI3, MLC2v). Indeed, neither human CD31 nor cytokeratin-positive cells were found in the grafts. Our data is corroborated by previous reports in different species [25,26,28,32]. Furthermore, we indirectly assessed hiPSC-CM proliferation in situ by calculating the percentage of Ki67- and PHH3-positive cells in the grafts. These proxies are limited approximations that do not guarantee that cells in the active cell cycle will complete the cytokinesis [45]. Despite the technical limitation, approximately 10% of the hKu80-positive cells were also Ki67 positive (data obtained from three rats), and 3% were also PHH3 positive (data obtained from five rats), percentages similar to those observed in previous reports [34,55]. In addition, Funakoshi et al. demonstrated in vitro that day 10 cells are twice more proliferative than day 20 and five times more proliferative than day 30 cells. These authors also tracked hiPSC-CM proliferation by Ki67 expression in situ up to six months after cell injection in a mice model of MI. hiPSC-CM proliferation was sharply reduced three months after injection [21]. We believe that hiPSC-CM-derived grafts may be growing in vivo. However, it is worth mentioning that, regardless of their proliferation capacity, the percentage of engrafted cells achieved in our study and others is still far from being translated into massive structural and functional improvements able to completely change myocardial infarction outcomes.

The grafts identified in this study displayed remarkable maturation in situ, demonstrated by a shift between MCL2a and MLC2v expression. hiPSC-CMs on day 11–15 of differentiation (the day on which these cells were injected) did not express MLC2v, a proxy for ventricular-like cell maturation. Thirty days later (41–45 days old cells), the expression of MLC2a was almost abolished, whereas MLC2v expression increased significantly in vivo. Human grafts expressed substantial amounts of Pan-cadherins and low amounts of Cx43 and Cav3 compared to the host tissue. Furthermore, the human cardiac tissue was vascularized by vWF-positive host blood vessels. These data are corroborated by previous reports in different species [22,25,26,33,34]. Based on previous reports assessing cardiac grafts up to six months after injection [21,22,33], we believe that more prolonged periods of post-treatment observation would also enhance understanding of the molecular and structural maturation of hiPSC-CM grafts in vivo.

Despite the limited expression of Cx43 and Cav3 [22,23,30], suggesting a limited graft/host tissue coupling, we demonstrated that the injection of hiPSC-CM promotes significant segmental contraction improvement. The segmental contraction results in overall cardiac function improvement in cell-treated rats. We genuinely believe that the human cardiac graft physical and electrical interactions with the host tissue are the primary mechanism of action behind the improved global contraction in the cell-treated rats. However, we cannot exclude alternative mechanisms based only on our approach. In addition to the direct graft/host interactions, previous authors have demonstrated that hiPSC-CMs could also release molecules with the potential to influence the microenvironment of cardiac healing [56,57]. We recognize this alternative hypothesis, but our findings do not give us substrate to assume anything in this direction.

In parallel to the maturation analysis in vivo, we performed flow cytometry analysis for MLC2a, MLC2v, Cx43, and Cav3 to monitor hiPSC-CMs maturation in vitro. The percentage of MLC2v-positive cells increased over time (up to day 50). Interestingly, in contrast with our data in vivo, MLC2a remains highly expressed in hiPSC-CMs for up to 50 days in culture. In addition, Cx43 and Cav3 were not expressed or only partially up-regulated in the population after 50 days in culture, respectively. Altogether, our in vivo and in vitro findings corroborate an enhancement of hiPSC-CMs maturation in situ with limited host/graft interaction.

Although we strove to work under clinically relevant conditions, it is worth listing some limitations to guide the interpretation of our results. Although PluriCell Biotech disposes of other well-characterized hiPSC lines in its portfolio, we used only one cell line showing superior reproducibility regarding cardiac differentiation [37]. We cannot exclude the possibility that other cell lines behave slightly differently in this context. On the other hand, future clinical applications might incorporate the concept of off-the-shelf therapy, where the use of a universal non-immunogenic cell would be desirable. Second, a xenotransplant always imposes additional immunogenic complexities. We implemented a very standard cyclosporine A protocol to mitigate the rejection of the human cells. Blood samples for dosing CsA were collected during the study. Due to transportation issues, these samples were lost, precluding an acceptable dosage of CsA over time. CsA can affect cardiac function through various mechanisms [58,59]. One of our SHAM animals suddenly died with clear signs of intoxication (e.g., cachexia, necrosis of abdominal skin and muscle). Additionally, the SHAM group displayed impaired LVEF compared to healthy rats (which did not receive immunosuppressants). We cannot exclude that survivors also had some degree of CsA-related toxicity affecting their overall cardiac function. In addition, our histopathological evaluation of immune rejection shows a slight trends towards higher inflammatory infiltration in the hearts of CELL-treated rats. Others have similarly reported this trend in different species and treatments [12,25,30,33,60,61].

We intentionally used female rats for this study. Historically, female rodents show higher survival rates post-MI induction [62,63,64]. Furthermore, the meaning of gender differences in cardiovascular diseases is still very controversial [65,66,67,68]. The use of both genders would benefit future preclinical studies in exploiting systematic differences within cell therapy.

Notably, our rats were exclusively treated with hiPSC-CMs without concurrent well-established drug-treatments such as beta-blockers and angiotensin-converting enzyme inhibitors, which can be a beneficial combination to use with a cell-therapy [12]. Importantly, our 30 days follow-up period is short for the observation of more sustained improvements in cardiac function and graft maturation. Finally, based on the previous well-established reports regarding graft-related arrhythmias [25,26,29,30,33,34] and the rat model’s intrinsic limitations to access such a complex interplay between host and graft, we deliberately avoided assessing the functional coupling of hiPSC-CM grafts in this study. To overcome this limitation, we are already working with pig models of MI. We will study the coupling of cells with the host tissue in this model that displays anatomopathological, metabolic, and even cellular conditions more similar to humans [12,24,25].

Altogether, our findings support the use of early-stage hiPSC-CMs as an alternative therapy to regenerate segments of the myocardium in infarcted rats. The present work demonstrated that hiPSC-CMs on days 11–15 of differentiation, injected seven days after MI induction, (1) ameliorate overall cardiac function through segmental contraction enhancement; (2) display clear signs of maturation in vivo toward a ventricular-like phenotype; (3) preserving some proliferation capacity. Moreover, these human grafts expressed interactive proteins (massive amounts of Pan-cadherin, low amounts of Cx43 and Cav3) supporting a host/graft limited interaction. Finally, (4) we found host circulation in the human grafts in all the animals, and (5) we did not find significant immune rejection response nor abnormal tissue formations in or out of the heart. This proof-of-concept article is an additional resource in the endeavor towards developing a clinical-grade regenerative cell-based therapy to treat patients affected by degenerative cardiac diseases such as myocardial infarction.

## 4. Material and Methods

All procedures and analyses were carried out by operators blinded to the experimental groups. Codes were revealed only when all the analyses were completed. Further detailed information regarding the following experimental procedures and materials is presented in a Supplementary Information document.

### 4.1. Immunosuppression Confidently Preserves Human Cardiac Grafts from Rejection or Ectopic Cellular Formations

This investigation agrees with the Declaration of Helsinki and the study protocol was approved by the Ethics Committee for Medical Research on Human Beings of the Institute of Biomedical Sciences from the University of Sao Paulo (#2.009.562) and the Committee on Animal Research and Ethics (CARE) at the Federal University of Rio de Janeiro (UFRJ) protocol #117/18. Signed informed consent was obtained from the cell donor. Animal experimentation and care agree with the ARRIVE (Animals in Research: Reporting In Vivo Experiments) [69].

### 4.2. hiPSC-CM Differentiation

Fresh (cells that have never been frozen) early-stage hiPSC-CMs (on days 11–15 of differentiation) were derived from the ACP5 hiPSC (a PluriCell Biotech hiPSC line). Briefly, hiPSCs were generated from healthy-donor erythroblasts using an episomal reprogramming system [37]. Cardiomyocytes were differentiated using a monolayer-based protocol previously described [37].

### 4.3. hiPSC-CM Characterization: Flow Cytometry and Immunofluorescence

hiPSC-CMs were characterized by flow cytometry and immunofluorescence assays. Detailed lists of antibodies (Table S3), reagents (Table S4), and descriptive protocols are provided in the Supplementary Information document.

### 4.4. Myocardial Infarction Induction

Two-month-old adult female Wistar rats (150–200 g body weight) were subjected to surgical procedure to induce myocardial infarction (day 0). The left anterior descending coronary artery (LAD) was permanently occluded as previously described [38].

### 4.5. Immunosuppression

Except for the healthy CTRL animals, rats were treated with 20 mg/kg of Cyclosporine A (Sandimmun IV, Novartis, Switzerland) [70,71,72,73,74] daily (2 doses of 10 mg/kg every 12 h), via intraperitoneal injection from day 2 to euthanasia.

### 4.6. Early-Stage hiPSC-CM Priming and Intramyocardial Injection

hiPSC-CMs were primed by heat-shock [23] before the intramyocardial injection. On day 7 of the experimental timeline, 10 million cells (on days 11–15) were suspended in 150 uL of a pro-survival solution supplemented with approximately 0.4 mg of GelTrex (ThermoFisher) [27] and directly injected into the infarcted rats’ left ventricle through a second surgical thoracotomy. Injections were split into three sites inside of and surrounding the scar tissue. Placebo animals were injected with the pro-survival solution supplemented with GelTrex without cells.

### 4.7. Echocardiography, Randomization, and Exclusion Criteria

Animals under inhalation anesthesia were subjected to echocardiography on days 6 (pre-) and 37 (post-injection). Parasternal images were captured using a Vevo^®^ 2100 ultrasound equipment (Fujifilm VisualSonics, Inc., Toronto, Canada) using a MS250 transducer (13–24 MHz). Analyses were performed using Vevo LAB (Fujifilm VisualSonics, Inc., Toronto, Canada). American Society of Echocardiography recommendations [75] were followed for the data analysis and interpretation. On day 6, MI-induced rats were randomized by LVEF in two balanced groups. Moreover, animals (placebo or treated) with less than 20% LVEF impairment (at baseline) compared to control animals (LVEF above 55%) were excluded from the final analyses (functional and histopathological).

### 4.8. Euthanasia and Tissues Sampling

On day 37, after the final echocardiography and after deep anesthesia, animals were euthanized by potassium chloride overdose. Thoracic and abdominal regions were photographed and the organs of interest (lung, liver, kidney, spleen, and left cerebral hemisphere) were collected and stored at –80 °C. Hearts were fixed using 4% paraformaldehyde (PFA) for further histopathological and molecular analyses.

### 4.9. Histology, Immunohistochemistry (IHC), and Immunofluorescent (IF) Assays

The hearth was removed from the thoracic region after euthanasia. The tissue was cross-sectioned in the middle region, washed with 1× PBS, and PFA-fixed for histological, immunohistochemistry (IHC), and immunofluorescent (IF) staining (antibodies are listed in Table S1). Details are provided in the Supplemental Materials. Immunofluorescence and immunohistochemistry micrographs were obtained using TissueFAXS slide scanner (TissueGnostics, Austria), confocal microscope LSM 800 (Zeiss-Germany), and a conventional fluorescence microscope Axio Imager 2 (Zeiss-Germany). The analyses were performed using ImageJ.

### 4.10. Biodistribution

To evaluate the migration and survival of hiPSC-CMs in other tissues after injection in the heart human, mitochondrial DNA were searched for in kidney, lung, brain, liver, and spleen tissue using RT-PCR and RT-qPCR as previously described by Liu et al. [26].

### 4.11. Statistical Analysis

Results are expressed as mean ± standard error of the mean, expect where indicated. One- or two-way analysis of variance (ANOVA) with Bonferroni posthoc test or unpaired Student’s t-test were utilized to compare groups as appropriate. All statistical analyses were performed using GraphPad Prism 8.0 (GraphPad Software Inc., CA, USA). *p*-values < 0.05 were considered significant. For *p* < 0.05 = *; *p* < 0.001 = **; *p* < 0.0005 = *** and *p* < 0.0001 = ****, expect where indicated.

## Figures and Tables

**Table 1:** Overall mortality and excluded animals by phase and group.

Status	Phase	N#/ Phase	% (From Total)	Cause	Group	N#/ Group	% (From Total)
Dead	MI induction	1	2.4%	Irreversible fibrillation	MI	1	2.4%
	1–6 days after MI induction (pre-echocardiography)	2	4.8%	Anesthetic overload	MI	1	2.4%
				Sudden death *	MI	1	2.4%
	Baseline echocardiogram (day 6)	2	4.8%	Anesthetic overload	MI	2	4.8%
	Injections procedure	6	14.3%	Cardiorespiratory arrest	PSC	3	7.1%
					CELL	3	7.1%
	1–30 days after injection (pre-final echocardiography)	6	14.3%	Sudden death *	SHAM	1	2.4%
					PSC	3	7.1%
					CELL	2	4.8%
	Final echocardiogram (day 37)	0	0.0%	-		0	0.0%
Live	LVEF cutoff (based on CTRLs)	4 **	9.5%	20% impairment vs. CTRL animals at baseline	PSC	2	4.8%
					CELL	2	4.8%
	Completed follow-up and used for further analysis	21	50.0%	-	CTRL	4	9.5%
				-	SHAM	4	9.5%
				-	PSC	6	14.2%
				-	CELL	7	16.7%
Total		42	100%			42	100%

* These animals were found dead in their cages. ** These animals were excluded from subsequent analysis.

## Data Availability

The data presented in this study are available on request from the corresponding author.

## Supplementary Materials

Supplementary methods, Tables, and Figures have been incorporated into a single PDF file. The following PDF is available online at https://www.mdpi.com/article/10.3390/jpm11050374/s1.
